# A data set with kinematic and ground reaction forces of human balance

**DOI:** 10.7717/peerj.3626

**Published:** 2017-07-27

**Authors:** Damiana A. dos Santos, Claudiane A. Fukuchi, Reginaldo K. Fukuchi, Marcos Duarte

**Affiliations:** 1 Biomedical Engineering Program, Universidade Federal do ABC, São Bernardo do Campo, São Paulo, Brazil; 2 Neuroscience and Cognition Graduate Program, Universidade Federal do ABC, São Bernardo do Campo, São Paulo, Brazil

**Keywords:** Biomechanics, Posturography, Stabilography, Posture, Motor control

## Abstract

This article describes a public data set containing the three-dimensional kinematics of the whole human body and the ground reaction forces (with a dual force platform setup) of subjects who were standing still for 60 s in different conditions, in which the subjects’ vision and the standing surface were manipulated. Twenty-seven young subjects and 22 old subjects were evaluated. The data set comprises a file with metadata plus 1,813 files with the ground reaction force (GRF) and kinematics data for the 49 subjects (three files for each of the 12 trials plus one file for each subject). The file with metadata has information about each subject’s sociocultural, demographic, and health characteristics. The files with the GRF have the data from each force platform and from the resultant GRF (including the center of pressure data). The files with the kinematics contain the three-dimensional positions of 42 markers that were placed on each subject’s body and 73 calculated joint angles. In this text, we illustrate how to access, analyze, and visualize the data set. All the data is available at Figshare (DOI: 10.6084/m9.figshare.4525082), and a companion Jupyter Notebook presents programming code to access the data set, generate analyses and other examples. The availability of a public data set on the Internet that contains these measurements and information about how to access and process this data can potentially boost the research on human postural control, increase the reproducibility of studies, and be used for training and education, among other applications.

## Introduction

The ability to maintain an upright posture is a vital skill needed to perform most of our daily life activities, and this ability is a complex task that is dependent on a rich and fine integration of the central nervous system with the sensory and musculoskeletal systems. One of the most basic and easily observable facts related to the maintenance of an upright posture is that our body sways while we stand even if we try to stay as still as possible. To quantify the amount a person sways, the displacements of the segments of the body can be measured, and these measurements can be used to estimate the displacement of the body’s center of gravity (COG). A physical quantity related to and that is simpler to measure than COG is the body’s center of pressure (COP), which expresses the position of the resultant reaction force applied to the body (to our feet) at the ground’s surface. As we sway while standing, both COG and COP positions vary over time, and these physical quantities are the most commonly used quantities to quantify a person’s postural sway in both clinical and research contexts (Duarte & Freitas, 2010; Visser et al., 2008; Winter, Patla & Frank, 1990). However, the exact nature of the control mechanisms that allow humans to maintain their balance and the extent of the information that can be extracted from a person’s COG and COP positions are still under investigation.

A publicly available data set containing measurements of the COG and COP positions of standing subjects, which are measurements that require a well-equipped laboratory, would allow researchers to test different hypotheses about human postural control, to test different algorithms to extract meaningful information from these measurements, and generally contribute to greater reproducibility of studies that utilize these data sets. There are already a few data sets of this nature available in the literature, but these data sets contain limited data. The available data sets include: measurements of the displacement of a marker at a subject’s shoulder (as an estimation of whole body postural sway) for 27 subjects during quiet standing, which originated from a study about the effects of vibration on human balance (Priplata et al., 2003); measurements of the COP position of 38 subjects during quiet standing, which originated from a study about the effects of different types of visual stimuli on human balance (Perakakis et al., 2012); measurements of the COP position of 43 subjects during quiet standing, which originated from a study about the relationship between postural sway and motion sickness (Laboissiere et al., 2015); and measurements of the COP position of nine subjects during quiet standing, which originated from a study about human postural control (Funato et al., 2016). All these data sets contain companion data of studies that investigated a specific research question, so these data sets naturally have limited value for use in studies on different topics. Recently, we made available a data set of human balance evaluations with the explicit intention of generating public data for use by others (Santos & Duarte, 2016). This data set contained the COP positions of 163 subjects who were standing still in four different conditions (three trials per condition), in which vision and the standing surface were manipulated; this set also contained other evaluations of each subject’s balance and detailed information about each subject’s sociocultural, demographic, and health characteristics. Although this data set could potentially fulfill the needs of a broader audience, it still has limitations that prevent the data from addressing some important questions about human postural control.

Most studies on balance control have been based on data acquired from a single force platform setup. However, a person’s COP during regular bipedal standing is the net COP of the resultant forces from each foot, and these distinct COP values can only be measured using a force platform under each foot (Winter, 1995), which might be useful for a greater understanding of human postural control. Another question that requires more data is how humans coordinate the movement of their segments to control their balance while standing. This question has been investigated since the classical observation of the ankle and hip strategies that are used to control balance after a perturbation (Horak & Nashner, 1986).

We now seek to contribute a different public data set with more quantitative measurements to help investigate questions related to human balance. Therefore, the aim of the present article is to describe a public data set with a rich quantitative evaluation of human balance, and in this data set, we measured the three-dimensional (3D) kinematics of the whole human body and the ground reaction forces (GRFs) (with a dual force platform setup) of subjects standing during different vision and surface conditions. We also demonstrate how to access the data set and perform some basic data analysis.

## Methods

The data collection was performed in the Laboratory of Biomechanics and Motor Control at the Federal University of ABC, Brazil. The entire data collection for each subject was performed in a single session, and each session lasted one hour. A pilot study with four subjects was conducted to establish the experimental protocol of this study. The data of these four subjects is not included in this data set. This study was approved by the local ethics committee of the Federal University of ABC (CAAE: 53063315.7.0000.5594), and all subjects signed a consent form prior to the data collection.

### Subjects

A convenience sample of 49 subjects was recruited to participate in this study, and they were assigned to one of two groups according to their age: young (15 males and 12 females who were between 18 and 40 years old) and old (11 males and 11 females who were 60 years old or older). The subjects were recruited by word of mouth from local communities and included students from the university, the local neighborhood, and a community center for older adults. The subjects were interviewed to collect information about their demographic characteristics, sociocultural characteristics, and overall health condition.

### Data acquisition

**Figure 1:**
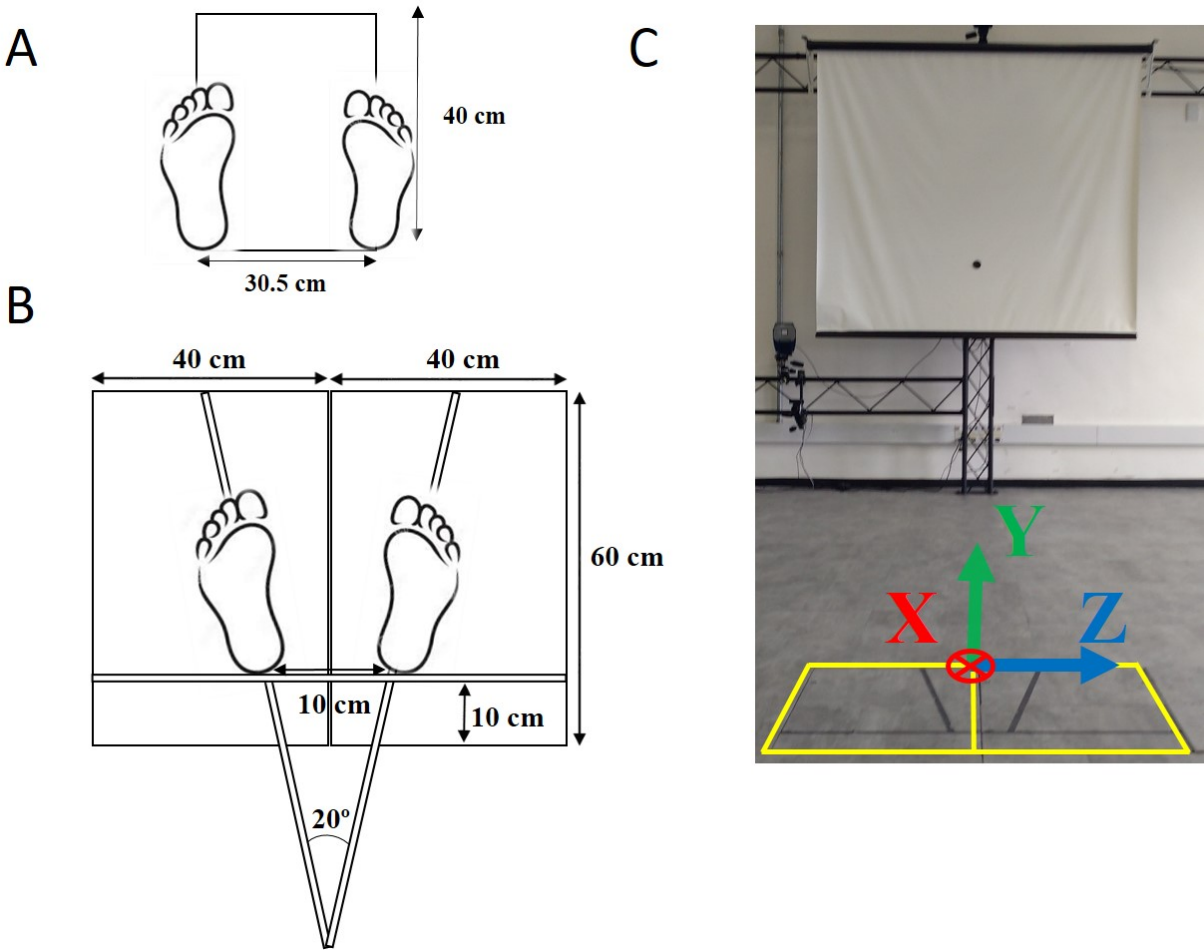
(A) Lines on the floor used for the alignment of a subject’s feet in a standardized position during the calibration trial. (B) Lines for the subject’s feet placement on the force platforms or on the foam blocks during the quiet standing trials. (C) Data collection room for the stabilography (note the 5-cm black target at the wall 4.35 m ahead), the two force platforms (marked in yellow), and the laboratory coordinate system convention (XYZ vectors). X denotes the anterior–posterior direction (+ is forward), Y denotes the vertical direction (+ is upward), and Z denotes the mediolateral direction (+ is to the right side of the subject).

Data about each subject’s balance was collected during bipedal quiet standing, which followed the same standing conditions described by Santos & Duarte (2016), but in the present study, we used a dual force platform setup and recorded each subject’s full-body 3D kinematics using a motion capture system. The subjects were evaluated while standing still for 60 s under four different conditions, in which vision and the standing surface were manipulated: on a rigid surface with eyes open, on a rigid surface with eyes closed, on an unstable surface with eyes open, and on an unstable surface with eyes closed. Each condition was performed three times, and the order of the conditions was randomized for each subject. The examiner performed the randomization before the data collection using a computerized random number generator. For the rigid surface conditions, the subjects stood on two 40 × 60 cm force platforms (OPT400600-1000; AMTI, Watertown, MA, USA) under each foot, and for the unstable surface conditions, the subjects stood on two 6-cm high foam blocks (Balance Pad; Airex AG, Sins, Switzerland), one of which was placed on each force platform. In all conditions, the subjects were required to stand barefoot and as still as possible with their arms at their sides. In the eyes-open conditions, each subject looked at a 5 cm round black target placed at the subject’s eye height on a wall 4.35 m in front of the subject. In the eyes-closed conditions, the subjects were first instructed to look at the target with eyes open, find a stable and comfortable posture given the requirements, and close their eyes. A few seconds later, the data acquisition started. For all the trials, the subject’s feet were placed with an angle of 20 degrees between them, and their heels were kept 10 cm apart by asking the subjects to stand on lines marked on the top of the force platforms and on the foam blocks (see Fig. 1). The trials were conducted in a 11.5 ×9.3 m room with white walls and adequate illumination (see Fig. 1).

**Figure 2:**
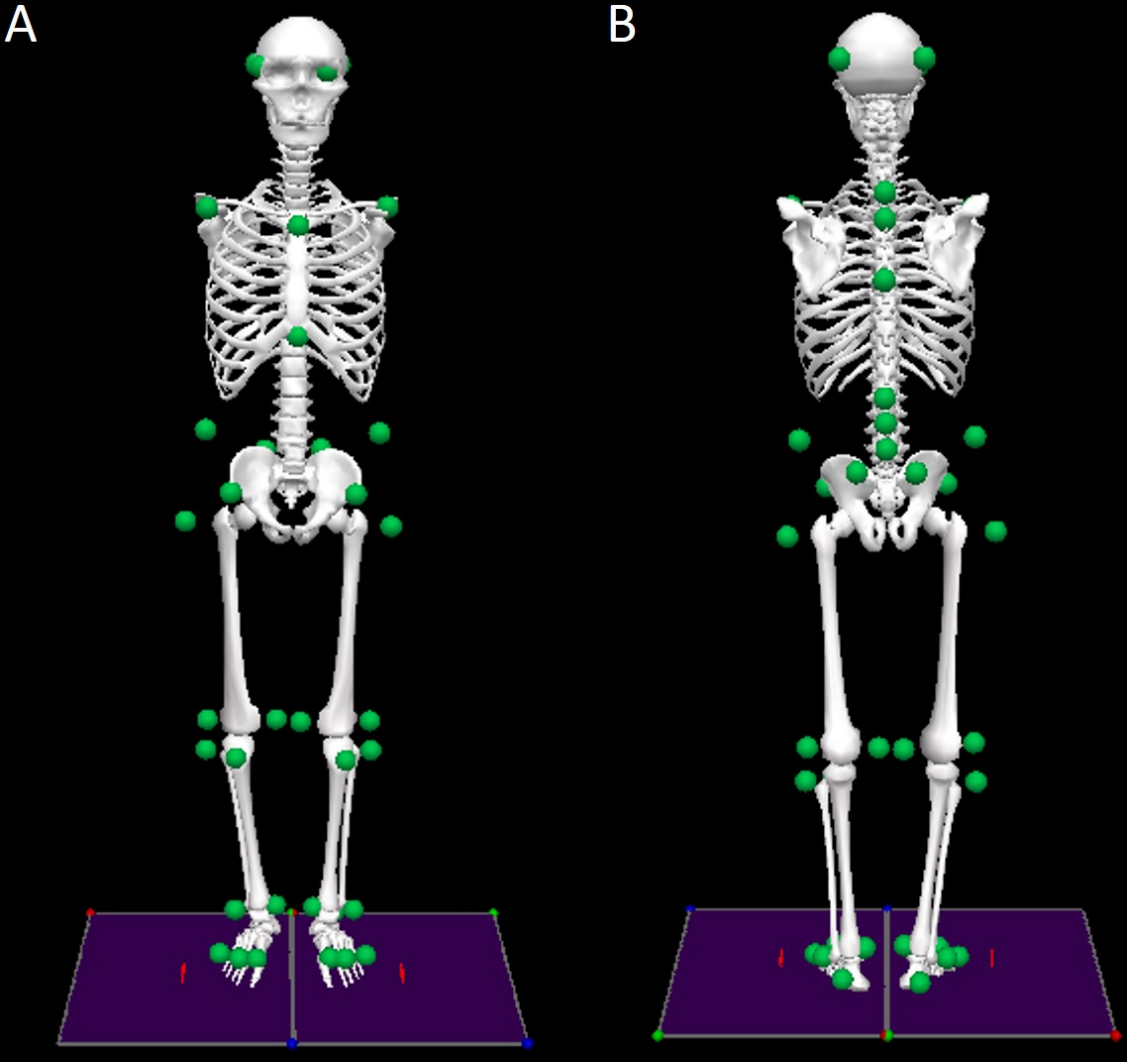
Front (A) and rear (B) views of the biomechanical model of the human body with the marker set convention (represented as green dots).

The full-body 3D kinematics of the subjects during the quiet standing trials were recorded using a motion capture system with 12 infrared cameras (Raptor-4, Motion Analysis, Santa Rosa, CA, USA). The displacement of the head, trunk, pelvis, and right and left feet, thighs, and shanks were tracked using a marker set model that combined the lower-body and upper-body models proposed by Leardini and collaborators (2011 and 2007) plus four markers on the head and two markers on the iliac crests. In total, 42 reflective markers were placed on the subject’s anatomical landmarks (see Fig. 2 and Table S1). Of note, the upper limbs were not tracked by the motion capture system because each subject was instructed to maintain the placement of his or her arms along his or her trunk during the trials.

The data acquisition of the GRF and 3D markers’ positions were performed at a sampling frequency of 100 Hz with the Cortex software version 5.3 (Motion Analysis, Santa Rosa, CA, USA). Using the Cortex software, all the data of each trial was exported to a file in the c3d format (https://www.c3d.org/).

#### Protocol

The following procedure was adopted for the data collection (based on Santos & Duarte, 2016):

1. The motion capture system was calibrated and the force platforms were zeroed according to the equipment’s manuals.
2. The researcher explained to each subject the process of data collection with the force plates and the motion capture system. The subject was informed that during the data collection, he or she would be monitored and that there should not be any verbal communication during the trials but that he or she could interrupt the data collection if desired and that assistance would be given if necessary.
3. After these explanations, the subject signed the informed consent form, and the subject was asked to stay barefoot, to change their clothes to tight-fitting clothes, and to place an elastic band around his or her head.
4. The researcher measured the subject’s body mass, height, and feet length.
5. The same researcher interviewed the subject to collect information about his or her sociocultural, demographic, and health characteristics.
6. The same researcher located 42 anatomical landmarks on the subject’s body using palpation, and passive reflective markers were placed on these landmarks with double adhesive tape (see Table S1 for the list of anatomical landmarks).
7. Standing calibration trial: Each subject was asked to perform a standing calibration trial for the kinematic measurement. A template was used to align the subject’s feet in a standardized position so that the long axes of the feet were parallel to the *X*-axis of the laboratory coordinate system (see Fig. 1). The subject was asked to stand still and look straight ahead. Then, the markers’ 3D coordinates were recorded for 3 s. After the acquisition was completed, the markers at the medial side of the right and left knees and ankles were removed because they could disturb the subject during the quiet standing trials.
8. Standing-still trials: The researcher instructed the subject how to stand on the force platforms according to the task (with open or closed eyes and standing on a rigid or unstable surface). The subject’s feet were positioned on the marks at the force platforms or on the foam blocks (see Fig. 1). The researcher instructed the subject to maintain the position of his or her arms along his or her body and to stand as still as possible. During the trials with the subject’s eyes open, the subjects were told to fix their gaze ahead on the round black target placed on the wall at the subject’s eye level. During the trials with eyes closed, the subjects were told to fix their gaze ahead at the same target, close their eyes when they felt ready, and only open them when the researcher indicated that the trial was over.
9. The researcher started the data collection around 5 s after the subject said he or she was ready.
10. At the end of the trial, the subject could rest (and sit if desired) for about one minute before the next trial.
11. If the subject was unable to complete the 60-s trial, the test was stopped, and that trial was immediately repeated up to two times if necessary. All subjects completed all trials.

### Data processing

Data processing, biomechanical modeling, analysis, visualization, and exportation of data to text files, was performed using custom programming implemented in the Visual3D software version 6.0 (C-Motion, Inc., Germantown, MD, USA) and in the SciPy Stack (https://www.scipy.org/) for the Python programming language. All the data for the GRF and marker positions was smoothed with a low-pass Butterworth filter with a 10 Hz cutoff frequency, fourth order, and zero lag.

#### Ground reaction force data

The COP position for each force platform was calculated according to the standard formula (Duarte & Freitas, 2010; Santos & Duarte, 2016), and the GRF data (including COP) were transformed from the coordinate systems of the two force plates into the laboratory coordinate system (the coordinate system created by the motion capture system) via transformation matrices using the Cortex software. As result, for each force platform, the data is presented as the X, Y, Z components of COP and force and the free moment of force (at the Y direction). The free moment of force is the moment around the normal to the force plate on the subject’s foot (Robertson et al., 2014).

From the GRF data of each force platform, we calculated the net (resultant) GRF and the net COP positions in the anterior–posterior (*x*-positive is anterior), vertical (*y*-positive is up), and mediolateral (*z*-positive is to the right) directions (according to the laboratory coordinate system):

\begin{eqnarray*}GRFNETx=RGRFx+LGRFx \end{eqnarray*}

\begin{eqnarray*}GRFNETy=RGRFy+LGRFy \end{eqnarray*}

\begin{eqnarray*}GRFNETz=RGRFz+LGRFz \end{eqnarray*}

\begin{eqnarray*}COPNETx= \frac{ \left( RCOPx\times RGRFy+LCOPx\times LGRFy \right) }{GRFNETy} \end{eqnarray*}

\begin{eqnarray*}COPNETy=0 \end{eqnarray*}

\begin{eqnarray*}COPNETz= \frac{ \left( RCOPz\times RGRFy+LCOPz\times LGRFy \right) }{GRFNETy} \end{eqnarray*}

where *L* and *R* represent the left and right force platforms with respect to the subject standing on them.

#### Kinematics data

The use of the anatomical-based protocols for marker placement and segment definition proposed by Leardini and collaborators (2011 and 2007) and the additional markers placed on each subject’s head allowed us to calculate two-dimensional projection angles based on four points for each joint and 3D Cardan angles with the following convention: The first rotation (flexion–extension) occurred in the mediolateral axis (*Z*-axis, perpendicular to the sagittal plane), the third rotation (internal/external rotation) was around the longitudinal axis (*Y*-axis, perpendicular to the transverse plane), and the second rotation (abduction/adduction) was around an axis perpendicular to the previous two axes, which, in the anatomic position, represents the anterior–posterior axis (*X*-axis, perpendicular to the frontal plane). This is the *Z*–*X*–*Y* convention, and it is frequently used to describe lower extremity rotations in the human body (Cappozzo et al., 1995). See Tables S2–S4 for a description of all the angles calculated. To estimate COG position based on the kinematic data, besides using the segments proposed by Leardini and collaborators (2011 and 2007), we altered the trunk of their model to a segment that included the head, arms, and trunk and used the mass and moment of inertia from the Dempster anthropometric model adapted by Winter (2009) for this segment and the other segments.

Next, we exemplify how to access, analyze, and visualize the data set. All the programming code used here for such examples is available as a Jupyter Notebook (see the Supplemental Information 2 and it can be viewed in https://github.com/demotu/datasets/blob/master/PDS/notebooks/PostureDataset.ipynb).

## Results

All the data is available at Figshare (DOI: 10.6084/m9.figshare.4525082); the data is stored in ASCII (text) format with tab-separated columns that can be downloaded as a single compressed file that is 6.93 GB large and that is made available under the CC0 1.0 license (https://creativecommons.org/publicdomain/zero/1.0/). The data set comprises a file with metadata plus 1,813 files with the GRF and kinematics data for the 49 subjects (three files for each of the 12 trials plus one file for each subject: 3 × 12 × 49 + 49 files).

### Metadata

The metadata file named PDSinfo.txt contains 29 fields about the conditions of the trials and information from the anamnesis of each subject. There are 12 rows for each subject in this file—one row for each of the 12 trials. In these 12 rows, the only columns that have rows with different values are the columns describing the trials. The content of all the other columns is simply repeated over the 12 rows. As a result, the PDSinfo.txt file has a header plus 588 rows with 29 columns. Here is the coding for the metadata (the first word identifies the name of the column in the header):

1. **Trial**: the file name of the stabilography trial (PDSXXYYZ, in which PDS stands for the project’s name (Posture Data Set); XX identifies the subject and varies from 01 to 49; YY identifies the stabilography condition and is either OR, OF, CF, or CR; and Z identifies the number of repetitions and varies from 1 to 3).
2. **Subject**: number of the subject (from 1 to 49).
3. **Vision**: visual condition (O: open eyes or C: closed eyes).
4. **Surface**: surface support condition (R: rigid or F: foam).
5. **Rep**: trial number (from 1 to 3).
6. **Age**: subject’s age in years.
7. **AgeGroup**: age group (Young: Age < 60; Old: Age ≥60).
8. **Gender**: gender (F or M).
9. **Height**: height in meters (measured with a calibrated stadiometer).
10. **Weight**: weight in kilograms (measured with a calibrated scale).
11. **BMI**: body mass index in kg/m^2^.
12. **FootLen**: foot length in cm (average of the two feet, measured with a calibrated paquimeter).
13. **DominantLeg**: preferred self-reported leg for kicking a ball (Right or Left).
14. **Nationality**: country where the subject was born.
15. **SkinColor**: self-reported skin color.
16. **Ystudy**: years of formal education.
17. **Footwear**: most common type of footwear the subject wears daily.
18. **Illness**: whether the subject had any illness at the time of the trials, as declared by the subject (Yes or No).
19. **Illness2**: type of illness of the subject (“No” if the subjects did not have any illness).
20. **Nmedication**: total number of medications the subject takes per day (if any).
21. **Medication**: name of the medication(s) the subject takes (“No” if the subject did not take any medication).
22. **Ortho-Prosthesis**: whether the subject wears any type of orthosis or prosthesis, as declared by the subject (Yes or No).
23. **Ortho-Prosthesis2**: name of the orthosis or prosthesis the subject wears (“No” if the subject did not wear any orthosis or prosthesis).
24. **Disability**: whether the subject has any disability, as declared by the subject (Yes or No).
25. **Disability2**: name of the disability of the subject (No if the subject did not present any disability).
26. **Falls12m**: how many unintentional falls the subject experienced in the last 12 months, as declared by themselves (from 0 to an unlimited upper limit).
27. **PhysicalActivity**: number of days per week the subject practiced physical activity (from 0 to 7).
28. **Sequence**: sequence of the four conditions of stabilography (e.g., OR, OF, CF, CR).
29. **Date**: date and time of the subject’s evaluation (yyyy-mm-dd hh: mm:ss.sss; 24-hour local time format).

### Subjects characteristics

The mean and range values for age, height, and mass of the 49 subjects at each age group are shown in Table 1. Seven (six elderlies and one young adult) of the subjects were classified as people with disabilities. Thirty subjects (20 elderlies and 10 young adults) reported to have an illness. The disability and illness of each subject are reported in the file with the metadata.

**Table 1:** Mean and range values for age, height, and mass of the subjects in the dataset.

Group	*N*	Age (years)		Height (m)		Mass (kg)	
		Mean	Range	Mean	Range	Mean	Range
Old	22	67.8	61.1–84.7	1.61	1.46–1.78	68.6	49.2–96.0
Young	27	28.1	21.8–37.9	1.71	1.53–1.92	70.3	44.3–126.3

### Ground reaction force data

Each text file with the GRF data is named by the corresponding trial (given at the first column of the metadata file) plus the suffix “grf” (e.g., PDS01OR1grf.txt is the file name for the first trial of the first subject). Each file has a header and 6,000 rows (60s × 100Hz) and 21 columns of data with six-digit numeric precision. The header refers to the signal at each column (see ‘Ground reaction force data’) and are (the first word identifies the name of the column in the header):

1. **Time**: Time in s.
2. **RGRF_X**: Right Ground Reaction Force (GRF) at the X direction in N.
3. **RGRF_Y**: Right GRF at the Y direction in N.
4. **RGRF_Z**: Right GRF at the Z direction in N.
5. **LGRF_X**: Left GRF at the X direction in N.
6. **LGRF_Y**: Left GRF at the Y direction in N.
7. **LGRF_Z**: Left GRF at the Z direction in N.
8. **GRFNET_X**: Resultant GRF at the X direction in N.
9. **GRFNET_Y**: Resultant GRF at the Y direction in N.
10. **GRFNET_Z**: Resultant GRF at the Z direction in N.
11. **RCOP_X**: Right Center of Pressure (COP) at the X direction in m.
12. **RCOP_Y**: Right COP at the Y direction in m.
13. **RCOP_Z**: Right COP at the Z direction in m.
14. **LCOP_X**: Left COP at the X direction in m.
15. **LCOP_Y**: Left COP at the Y direction in m.
16. **LCOP_Z**: Left COP at the Z direction in m.
17. **COPNET_X**: Resultant COP at the X direction in m.
18. **COPNET_Y**: Resultant COP at the Y direction in m.
19. **COPNET_Z**: Resultant COP at the Z direction in m.
20. **RFREEMOMENT_Y**: Right Free Moment at the direction Y in Nm.
21. **LFREEMOMENT_Y**: Left Free Moment at the direction Y in Nm.

**Figure 3:**
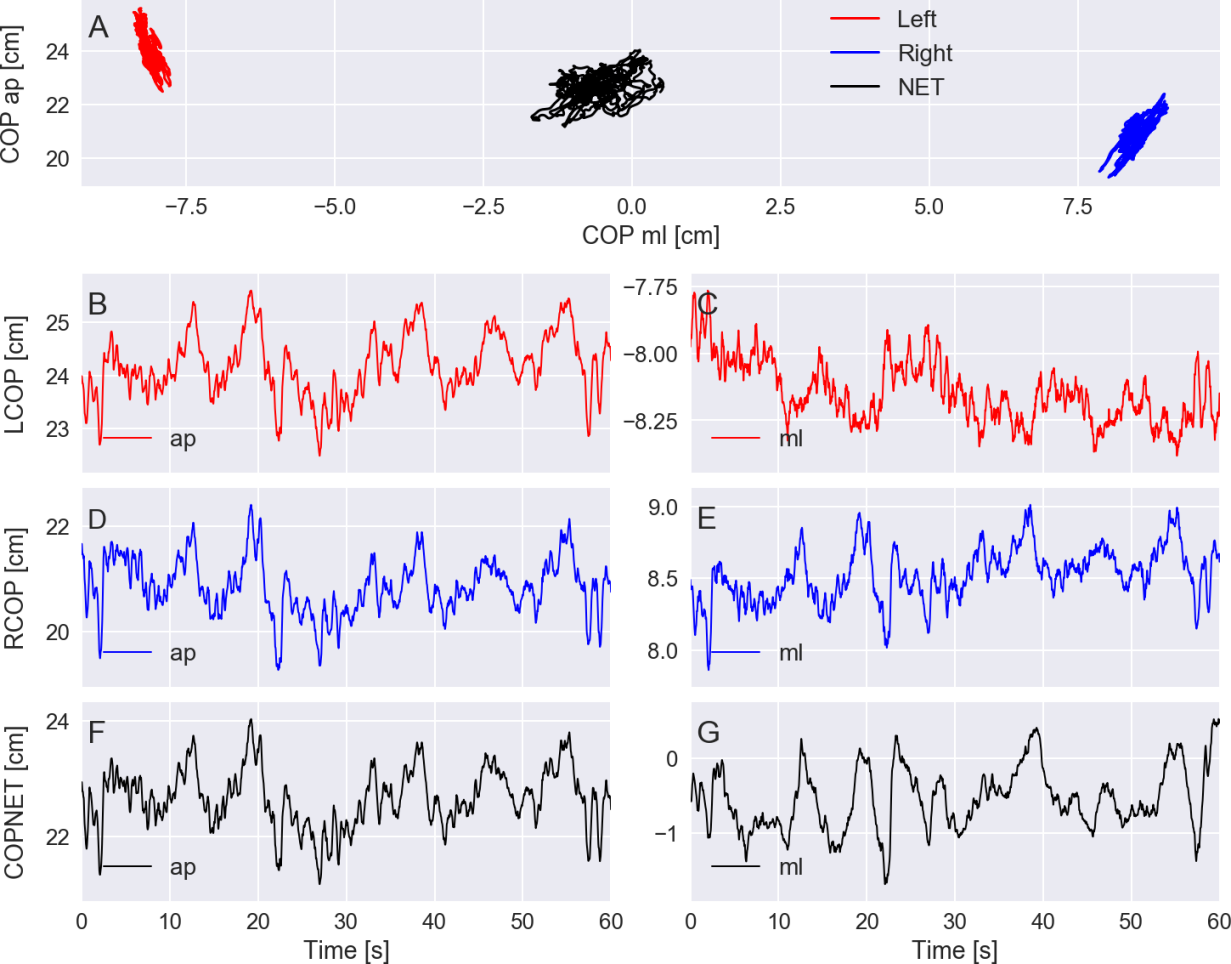
(A) exemplary plots of the center of pressure (COP) at the anterior–posterior (ap) direction versus the medio-lateral (ml) direction given by the left and right force platforms and the resultant COP (COPNET). (B–G) COP displacement at the ap and ml directions versus time for the left (LCOP) and right (RCOP) force platforms and for the COPNET. Trial PDS13CR1 (elderly subject standing with eyes closed on a rigid surface).

**Figure 4:**
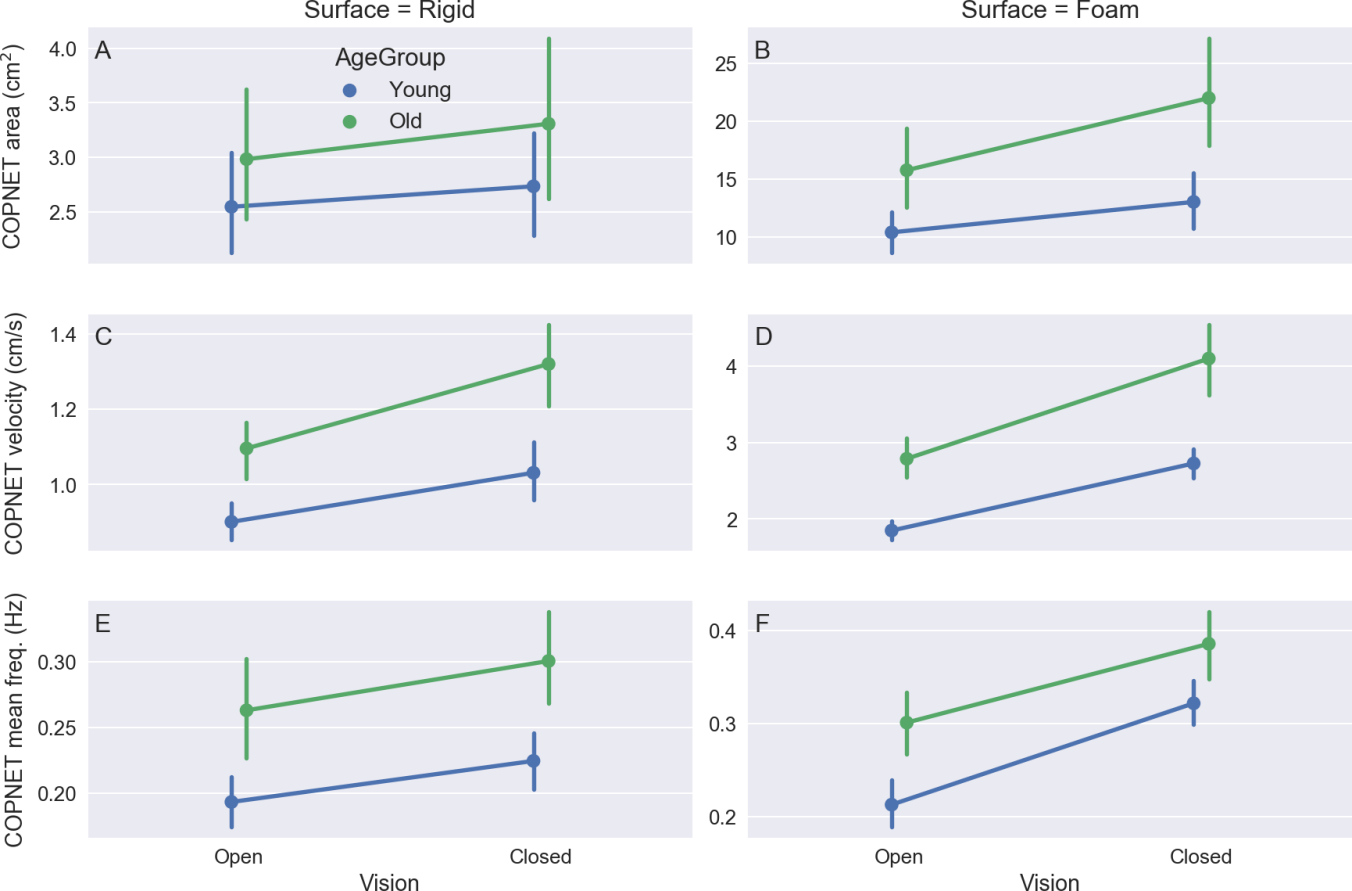
The mean and 95% confidence intervals across subjects of the variables COPNET area (A–B), the resultant COPNET velocity (C–D), and the resultant COPNET mean frequency (E–F) at the different visual and support surface conditions (color coded by age group).

For instance, Fig. 3 shows plots of the COP displacement on each force platform and the resultant COP from a trial of an elderly subject who was standing with eyes closed on a rigid surface. Some of the common variables to quantify the COP displacement during quiet standing used in the literature are (Duarte, 2015; Duarte & Freitas, 2010; Prieto et al., 1996): the area of the anterior–posterior COP versus the medio-lateral COP plot and the velocity and mean frequency of the COP displacement. The plots for these variables calculated for the resultant COP across the different standing conditions and grouped by the age group are shown in Fig. 4. In Fig. 4, the group of old subjects presents larger values than the group of young subjects for the calculated COP variables, and both groups present larger values in more challenging conditions (i.e., open vs. closed eyes and rigid vs. foam surface).

### Kinematics data

There are two kinds of files for the kinematics of each trial: a file with the markers and COG positions and a file with the segment and joint angles. Each of these files is named by its corresponding trial (given at the first column of the metadata file) plus the suffix “mkr” for the position data or the suffix “ang” for the angle data. Each file has a header and 6,000 rows (60 s ×100 Hz) of data with six-digit numeric precision. The file with the markers and COG positions has 130 columns (a time vector plus the X, Y, Z coordinates of 42 markers and of the COG). The file with the angles has 74 columns (a time vector plus 19 columns for the 19 planar angles plus 54 columns for the 18 3D Cardan joint angles). See Supplemental Information 1 for a description of the markers and angles names in the headers. In addition, for each subject, there is one extra file with the markers’ positions from the standing calibration trial for the kinematic measurement that contains 300 rows (3 s ×100 Hz) of data with six-digit numeric precision. This file is named PDSXXstatic.txt, where XX is the number of the subject. With this file, along with the markers’ positions and the GRF files, a user of the data set can define a biomechanical model consistent with the marker set we used and calculate any kinematic and kinetic variable (e.g., to calculate the joint moment of force). The corresponding units are: time (s), marker and center of gravity position (m), and angle (°).

**Figure 5:**
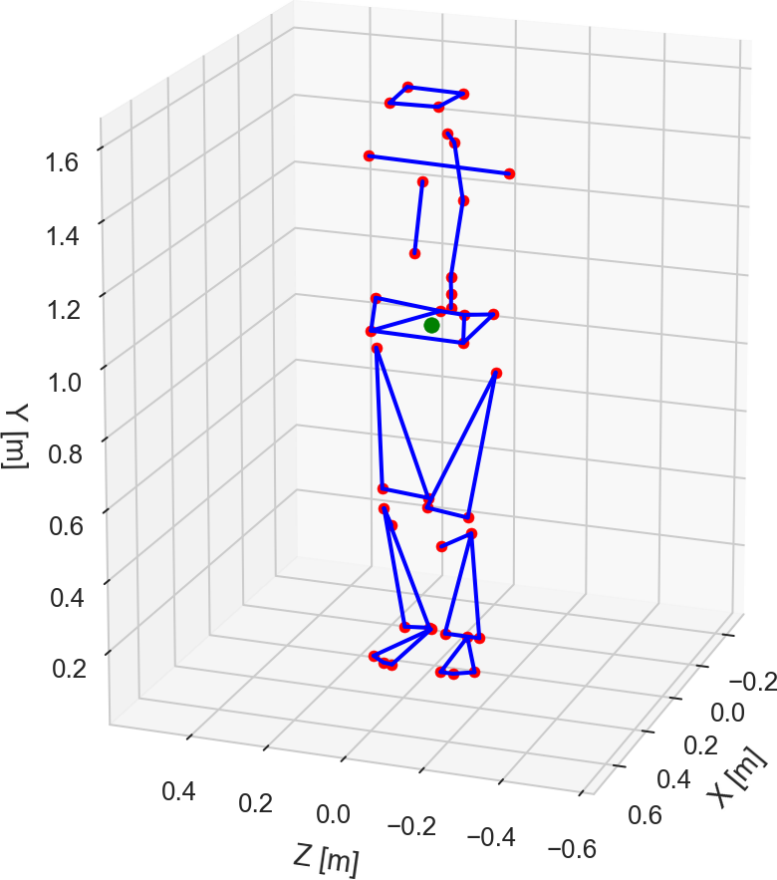
Average three-dimensional positions of the 42 markers (in red) and center of gravity (in green) during standing. Trial PDS13CR1 (elderly subject standing with eyes closed on a rigid surface).

**Figure 6:**
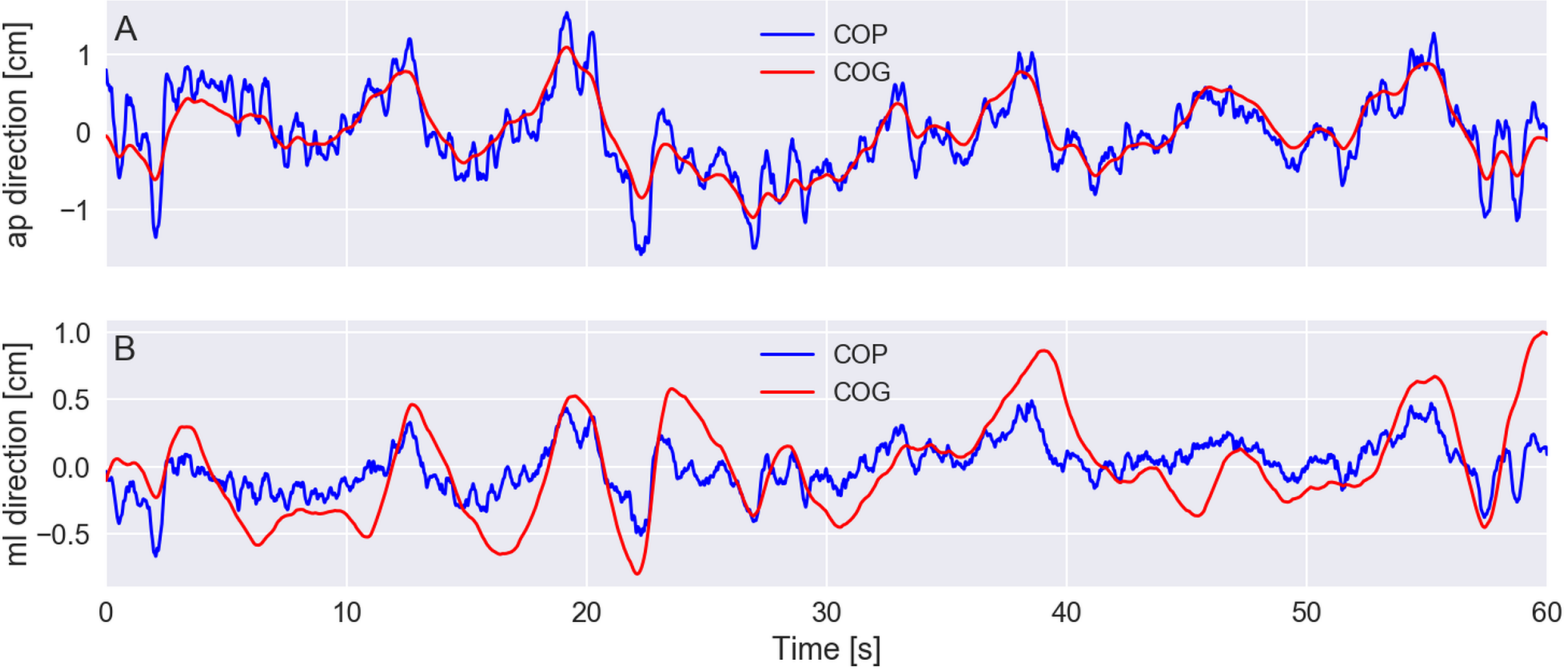
(A–B) exemplary plots of the center of pressure (COP) and center of gravity (COG) displacements at the anterior–posterior (ap) and medio-lateral (ml) directions. The COP and COG displacements had their corresponding mean values subtracted so that both signals have zero mean. Trial PDS13CR1 (elderly subject standing with eyes closed on a rigid surface).

For instance, Fig. 5 shows a plot with the average 3D positions of the markers and the COG from a trial of an elderly subject standing with eyes closed on a rigid surface. Figure 6 shows plots of the resultant COP and the COG displacement of the same trial. The COP and COG are very similar at the anterior–posterior direction but not as similar at the medio-lateral direction.

**Figure 7:**
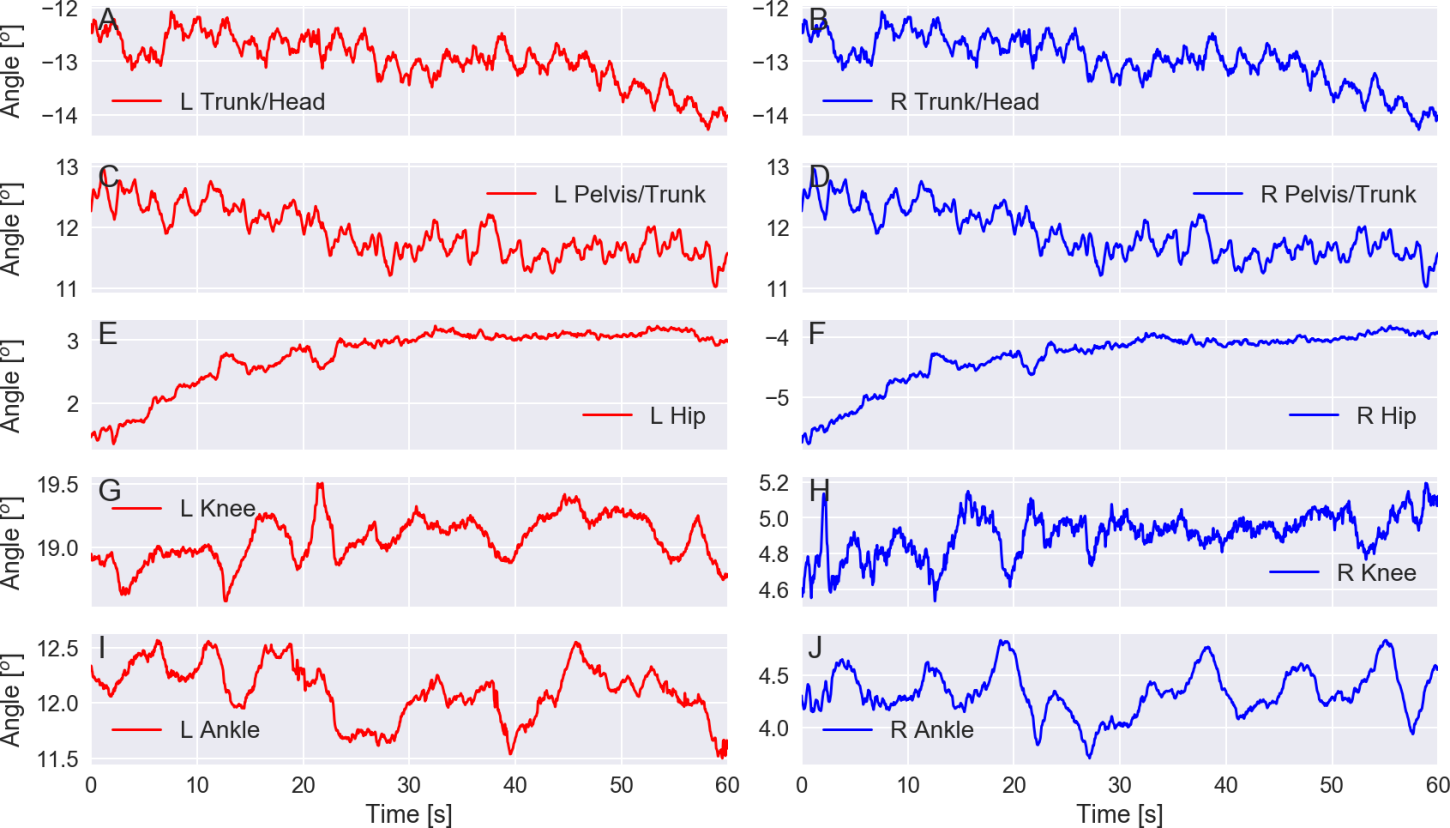
(A–J) exemplary plot of the Cardan angles at the sagittal plane (flexion/extension) for the trunk/head, pelvis/trunk, hip, knee, and ankle joints for the left (L) and right (R) sides. Trial PDS13CR1 (elderly subject standing with eyes closed on a rigid surface).

**Figure 8:**
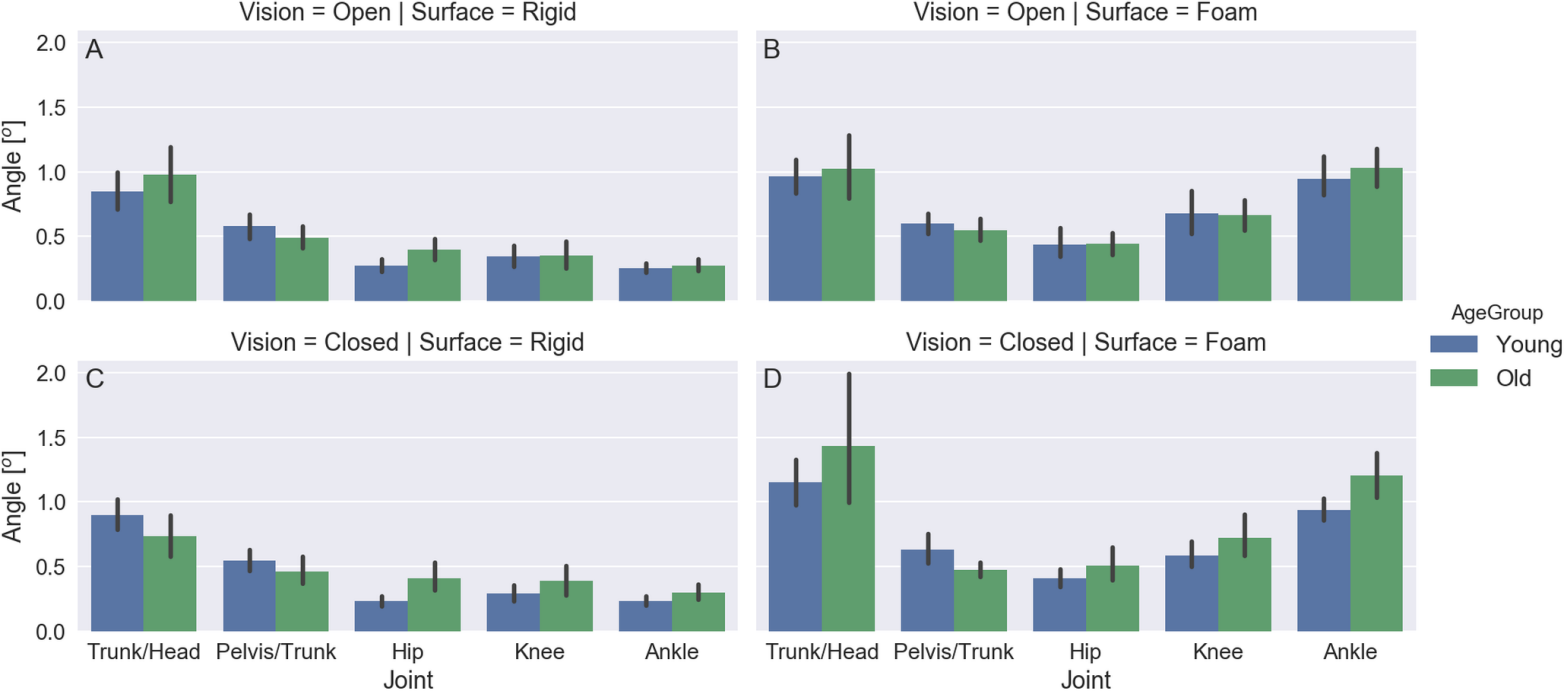
(A–D) mean and 95% confidence intervals across subjects of the amplitude range (maximum minus minimum) for the Cardan angles at the sagittal plane (flexion/extension) for the trunk/head, pelvis/trunk, hip, knee, and ankle joints at the different visual and support surface conditions (color coded by age group).

Figure 7 shows exemplary plots of the Cardan angles at the sagittal plane (flexion/extension) for the hip, knee, ankle, trunk/head, and pelvis/trunk joints during a trial of an elderly subject standing with eyes closed on a rigid surface. The angles for the right and left sides are very similar. Figure 8 shows plots for the mean and 95% confidence intervals across subjects of the amplitude range (maximum minus minimum) for these joint angles averaged between sides for all subjects. The joint angle ranges have similar values for both age groups and for the trials in which the subjects stood on a rigid surface; there is a bottom-up pattern for the joint angle range value that increases as the joint is furthest from the ground and closest to the head, but this pattern cannot be seen in the trials in which the subjects were standing on the foam surface.

## Discussion

The data set made publicly available at Figshare (DOI: 10.6084/m9.figshare.4525082) and described in this work includes data regarding the 3D kinematics of the whole human body and the GRFs (with a dual force platform setup) of 27 young and 22 older adults while standing in different conditions. We also made a file with metadata about the subjects’ sociocultural, demographic, and health characteristics available in the same data set.

In this article, we illustrated how this data can be accessed and explored; a companion Jupyter Notebook (see the Supplemental Information 2 and it can be viewed in https://github.com/demotu/datasets/blob/master/PDS/notebooks/PostureDataset.ipynb) presents the programming code to generate such analyses and other examples. The preliminary exploration of the data performed so far suggests that these subjects presented similar basic characteristics to the characteristics presented in different studies about human balance that employed similar methods (Freitas, Prado & Duarte, 2005; Prieto et al., 1996; Santos & Duarte, 2016; Teasdale, Stelmach & Breunig, 1991; Visser et al., 2008; Winter, 1995).

We studied a convenience sample of subjects who voluntarily participated in our study, so the sample might be unintentionally biased. Seven (six elderlies and one young adult) of the 49 subjects were classified as people with disabilities. For reference data on balance by healthy people, the data of these seven subjects should be excluded (this step is also exemplified in the companion Jupyter Notebook).

The key difference between the present data set and other data sets that contain information about human balance available in the literature is that the present data set provides full-body 3D kinematics and the GRF of each foot of the subjects during the standing trials in different visual and support surface conditions. These additional measurements are relevant to the scientific community considering the nature of human posture and the current research about human balance published in the literature. We hope that the availability of this public data set on the Internet and the included information about how to access and process this data boosts the research on human postural control, increases the reproducibility of studies on this topic, and is used for training and education, among other applications.

##  Supplemental Information

10.7717/peerj.3626/supp-1 Supplemental Information 1 Kinematic model information Supplementary material

10.7717/peerj.3626/supp-2 Supplemental Information 2 Jupyter Notebook and related functions

## Additional Information and Declarations

### Data Availability

The following information was supplied regarding data availability: Santos, Damiana A. dos; Fukuchi, Claudiane A.; Fukuchi, Reginaldo; Duarte, Marcos (2017): A data set with kinematic and ground reaction forces of human balance. figshare.
https://doi.org/10.6084/m9.figshare.4525082.v1. dos Santos DA, Fukuchi CA, Fukuchi RK, Duarte M. (2017) A data set with kinematic and ground reaction forces of human balance. PeerJ Preprints 5:e2865v1 https://doi.org/10.7287/peerj.preprints.2865v1.
