# Supplementary material for: A data set with kinematic and ground reaction forces of human balance
Source: PeerJ. 2017 Jul 27;5:e3626. doi: 10.7717/peerj.3626 (PMC5534162; doi:10.7717/peerj.3626)
Supplement: Supplemental Information 1 — Supplementary material [file peerj-05-3626-s001.pdf]

Supplementary material

This material is provided for: “A data set with kinematic and ground reaction forces of human balance”.

The marker set model used in this study is based on the work of Leardini and collaborators (2011; 2007). There is a tutorial of the Visual3D software for the IOR Full Body Model (http://c-motion.com/v3dwiki/index.php?title=Tutorial:_IOR_Gait_Full-Body_Model) which is close related to the present model we employed.

Table 1. Label, name, and description of the 42 markers used.

Label	Name	Description
**R.Front.Head /L.Front.Head**	Right/Left front head	Approximately over the right/left temple.
**R.Back.Head /L.Back.Head**	Right/Left back head	On the back of the head, roughly in a horizontal plane of the front head markers.
**R.Shoulder /L.Shoulder**	Right/Left shoulder	Right/Left Acromio-clavicular joint.
**C7**	7th cervical vertebrae	Spinous process of the seventh cervical vertebrae.
**T2**	2nd thoracic vertebrae	Second thoracic vertebrae.
**T7**	7th thoracic vertebrae	Seventh thoracic vertebrae.
**L1, L3, L5**	1st, 3rd, 5th lumbar vertebrae	First, third and fifth lumbar vertebrae.
**IJ**	Incisura jugularis	Deepest point of incisura jugularis.
**PX**	Xiphoid process	Xiphoid process (most caudal point of the sternum).
**R.ASIS/L.ASIS**	Right/Left anterior superior iliac spine	Anterior superior iliac spine.
**R.PSIS/L.PSIS**	Right/Left posterior superior iliac spine	Posterior superior iliac spine.
**R.Iliac.Crest/L.Iliac.Crest**	Right/Left iliac crest	Iliac crest.
**R.GRT/L.GRT**	Right/Left greater trochanter	Most lateral prominence of the greater trochanter.
**R.Knee/L.Knee**	Right/Left femoral epicondyle	Most lateral prominence of the lateral femoral epicondyle.
**R.Knee.Medial/L.Knee.Medial**	Right/Left medial femoral epicondyle	Most medial prominence of the medial femoral epicondyle.
**R.HF/L.HF**	Right/Left head of the fibula	Proximal tip of the head of the fibula.
**R.TT/L.TT**	Right/Left tibial tuberosity	Anterior border of the tibial tuberosity.
**R.Ankle/L.Ankle**	Right/Left lateral malleolus	Lateral prominence of the lateral malleolus.
**R.Ankle.Medial/L.Ankle.Medial**	Right/Left medial malleolus	Most medial prominence of the medial malleolus.
**R.Heel/L.Heel**	Right/Left heel	Aspect of the achilles tendon insertion on the calcaneous.
**R.MT1/L.MT1**	Right/Left 1st metatarsal head	Dorsal margin of the first metatarsal head.
**R.MT2/L.MT2**	Right/Left 2nd metatarsal head	Dorsal aspect of the second metatarsal head.
**R.MT5/L.MT5**	Right/Left 5th metatarsal head	Dorsal margin of the fifth metatarsal head.

Table 2. Label and name of the calculated angles.

	Planar angles		3D Cardan angles
**Label**	**Name**	**Label**	**Name**
**Sp5_Sp4_FE**	Sp5_Sp4 Flexion/Extension	**R/LHe_Lab_Angle**	Right/Left Head_Lab Angle
**Sp4_Sp3_FE**	Sp4_Sp3 Flexion/Extension	**R/LTh_Lab_Angle**	Right/Left Thorax_Lab Angle
**Sp3_Sp2_FE**	Sp3_Sp2 Flexion/Extension	**R/LPel_Lab_Angle**	Right/Left Pelvic Angle
**Sp2_Sp1_FE**	Sp2_Sp1 Flexion/Extension	**R/LHe_Th_Angle**	Right/Left Head_Thorax Angle
**Sp1_Pel_FE**	Sp1_Pelvis Flexion/Extension	**R/LTh_Pel_Angle**	Right/Left Thorax_Pelvis Angle
**R/LSp5_Sp4_LB**	Right/Left Sp5_Sp4 Lateral Bending	**R/LA_Th_Transl**	Right/Left Acromium_Thorax Translation
**R/LSp4_Sp3_LB**	Right/Left Sp4_Sp3 Lateral Bending	**R/LHip_Angle**	Right/Left Hip Angle
**R/LSp3_Sp2_LB**	Right/Left Sp3_Sp2 Lateral Bending	**R/LKnee_Angle**	Right/Left Knee Angle
**R/LSp2_Sp1_LB**	Right/Left Sp2_Sp1 Lateral Bending	**R/LAnkle_Angle**	Right/Left Ankle Angle
**R/LSp1_Pel_LB**	Right/Left Sp1_Pelvis Lateral Bending		
**R/LSh_Th_LB**	Right/Left Shouder_Thorax Lateral Bending		
**R/LSh_Th_AR**	Right/Left Shoulder_Thorax Axial Rotation		

Table 3. Convention for the planar angles.

Label	Convention
**Sp5_Sp4_FE**	Flexion (+)/Extension (-)
**Sp4_Sp3_FE**	Flexion (+)/Extension (-)
**Sp3_Sp2_FE**	Flexion (+)/Extension (-)
**Sp2_Sp1_FE**	Flexion (+)/Extension (-)
**Sp1_Pel_FE**	Flexion (+)/Extension (-)
**RSp5_Sp4_LB**<br>**LSp5_Sp4_LB**	Right (-)/Left (+)<br>Right (+)/Left (-)
**RSp4_Sp3_LB**<br>**LSp4_Sp3_LB**	Right (-)/Left (+)<br>Right (+)/Left (-)
**RSp3_Sp2_LB**<br>**LSp3_Sp2_LB**	Right (-)/Left (+)<br>Right (+)/Left (-)
**RSp2_Sp1_LB**<br>**LSp2_Sp1_LB**	Right (-)/Left (+)<br>Right (+)/Left (-)
**RSp1_Pel_LB**<br>**LSp1_Pel_LB**	Right (-)/Left (+)<br>Right (+)/Left (-)
**RSh_Th_LB**<br>**LSh_Th_LB**	Right (-)/Left (+)<br>Right (+)/Left (-)
**RSh_Th_AR**<br>**LSh_Th_AR**	Right (-)/Left (+)<br>Right (+)/Left (-)

Table 4. Convention for the tri-dimensional Cardan angles.

Label	Convention
**RHe_Lab_Angle**	Flexion/Extension: Anterior (+)/Posterior (-)<br>Lateral Bending: Right (-)/Left (+)<br>Rotation: Right (-)/Left (+)
**LHe_Lab_Angle**	Flexion/Extension: Anterior (+)/Posterior (-)<br>Lateral Bending: Right (+)/Left (-)<br>Rotation: Right (+)/Left (-)
**RTh_Lab_Angle**	Flexion/Extension: Anterior (+)/Posterior (-)<br>Lateral Bending: Right (-)/Left (+)<br>Rotation: Right (-)/Left (+)
**LTh_Lab_Angle**	Flexion/Extension: Anterior (+)/Posterior (-)<br>Lateral Bending: Right (+)/Left (-)<br>Rotation: Right (+)/Left (-)
**RPel_Lab_Angle**	Flexion/Extension: Anterior (+)/Posterior (-)<br>Lateral Bending: Right (-)/Left (+)<br>Rotation: Right (-)/Left (+)
**LPel_Lab_Angle**	Flexion/Extension: Anterior (+)/Posterior (-)<br>Lateral Bending: Right (+)/Left (-)<br>Rotation: Right (+)/Left (-)
**RHe_Th_Angle**	Flexion/Extension: Anterior (+)/Posterior (-)<br>Lateral Bending: Right (-)/Left (+)<br>Rotation: Right (-)/Left (+)
**LHe_Th_Angle**	Flexion/Extension: Anterior (+)/Posterior (-)<br>Lateral Bending: Right (+)/Left (-)<br>Rotation: Right (+)/Left (-)
**RTh_Pel_Angle**	Flexion/Extension: Anterior (+)/Posterior (-)<br>Lateral Bending: Right (-)/Left (+)<br>Rotation: Right (+)/Left (-)
**LTh_Pel_Angle**	Flexion/Extension: Anterior (+)/Posterior (-)<br>Lateral Bending: Right (+)/Left (-)<br>Rotation: Right (-)/Left (+)
**R/LA_Th_Transl**	Anterior (+)/Posterior (-)<br>Right (+)/Left (-)<br>Up (+)/Down (-)
**R/LHip_Angle**	Flexion (+)/Extension (-)<br>Adduction (+)/Abduction (-)<br>Rotation: Internal (+)/External (-)
**R/LKnee_Angle**	Flexion (+)/Extension (-)<br>Adduction (+)/Abduction (-)<br>Rotation: Internal (+)/External (-)
**R/LAnkle_Angle**	PlantarFlexion (-)/DorsiFlexion(+)<br>Adduction (+)/Abduction (-)<br>Inversion (+)/Eversion (-)
